# Effects of cardiorespiratory fitness and weight status on knowledge of physical activity and fitness, attitude toward physical education, and physical activity

**DOI:** 10.1186/s12889-018-5176-4

**Published:** 2018-02-20

**Authors:** Senlin Chen, Xiangli Gu

**Affiliations:** 1 School of Kinesiology, Louisiana State University, 175C Huey P. Long Field House, Baton Rouge, LA 70803 USA; 2 Department of Kinesiology, Health Promotion, and Recreation, University of North Texas, 1921 Chestnut Street, Denton, TX 76203 USA

**Keywords:** Adolescents, Fitness, Obesity, Physical activity, Physical education

## Abstract

**Background:**

The purpose of this study was to examine the effects of cardiorespiratory fitness and weight status on knowledge of physical activity and fitness (PAF knowledge), attitude toward physical education (PE), and physical activity.

**Methods:**

A total of 343 middle school students participated in the study (Age: M/SD = 12.76/.94, ranging from 11 to 14 years old). PE Metrics™ was used to measure PAF knowledge, and *Attitude toward Physical Education Questionnaire* and *Youth Activity Profile* were used to measure attitude, physical activity and sedentary behavior. Fitness and weight status were assessed using FitnessGram and converted to in Healthy Fitness Zone (HFZ) or Not in HFZ.

**Results:**

Two-way multivariate analyses of covariance (MANCOVA; gender and grade as covariates) showed a significant group effect for cardiorespiratory fitness (Λ_Pilla_ = .07, *F*_4,255_ = 5.03, *p* = .001, ${\eta}_p^2$ = .07) but not for weight status (*p* = .57). PAF knowledge (*F*_1,258_ = 9.49, *p* < .01, ${\eta}_p^2$= .04), attitude (*F*_1,258_ = 4.45, *p* < .05, ${\eta}_p^2$= .02) and sedentary behavior (*F*_1,258_ = 6.89, *p* < .01, ${\eta}_p^2$= .03) all favored the HFZ group.

**Conclusions:**

The findings reinforce the importance of promoting cardiorespiratory fitness in middle school PE as students acquire attitude, knowledge, and behaviors needed for active-living.

## Background

Teaching students for a lifetime of physical activity participation is an ultimate goal of physical education (PE) in K-12 schools [1, 2]. However, youth physical activity level is low calling for promotive effort for active-living [3–6]. Similarly, only a half of the students in the United States have reached the standards of FitnessGram [7, 8]. Coinciding with inadequate physical activity is the decline of health-related physical fitness (cardiorespiratory fitness in particular) [9] and the high prevalence of childhood obesity [10]. Fitness education is needed, especially in middle school years, for students to pursue knowledge, skills, attitude, and behaviors related to active-living [11]. Cardiorespiratory fitness and body weight status are important correlates of health and academic outcomes. It is well-documented that higher cardiorespiratory fitness is associated with reduced mortality risk, whereas obesity is related to co-morbidities and mortality [12–14]. Furthermore, cardiorespiratory fitness may offset the negative influence of obesity on health outcomes [15, 16], as improvement in fitness among individuals with obesity may modify their health perspective even although they may not change their body composition. In addition, recent research has substantiated the linkage between fitness/physical activity with academic achievement, performance, or cognition [9, 17, 18]. In the adolescent population, cardiorespiratory fitness and body weight status are significant correlates of academic outcomes [17, 18]. In particular, fitness in boys and weight status in girls were significantly correlated with health status and academic achievement, regardless of behavioral backgrounds [18].

PE is a main channel through which students receive formal education about fitness, health-related knowledge, physical activity, and weight management [19, 20]. Despite the difficulty to change students’ fitness and weight status through one setting alone within a short period of time, school-based physical activity programming that uses PE as the hub through a coordinate approach may lead to short-term and long-term changes to physical activity level and wellness status [21]. The influences of fitness and weight status on engagement and learning in PE related settings are under-researched. Most of the studies that focused on fitness and weight status have regarded them as outcome variables. In these studies, PE-based interventions or school-based interventions in which PE was included as a component have demonstrated small to moderate effect on fitness and/or weight status [22, 23]. Fewer studies have examined fitness and weight status as independent variables. One study demonstrated that both cardiorespiratory fitness and weight status are significant correlates of self-reported physical activity in youth, but cardiorespiratory fitness seems a stronger enabling factor than weight status [24]. The less active/less fit adolescents are less likely to participate in diverse activities during adulthood due to lack of competence, limited activity options and fitness abilities, and lower level of physical activity behavior [25, 26]. Compared to youth with healthy weight, those who are overweight or obese are less active and more sedentary [27].

While it is important to equip students with competence (e.g. knowledge and skills) [19, 28], it is equally important to foster them a positive attitude toward PE and physical activity [20, 29] and promote physical activity (discourage sedentary behavior) through PE classes [6, 20, 21], as students navigate pathways toward lifetime physical activity. Prior research has shown that students’ attitude toward PE decline by age [3, 6, 29], lack a good understanding of physical activity or fitness knowledge [19, 30], and spend excessive time in sitting behaviors [5, 6]. The purpose of this study was to examine the effects of cardiorespiratory fitness and weight status on middle school students’ knowledge, attitude, and behaviors (i.e., physical activity and sedentary behavior). It was hypothesized that students with more desirable cardiorespiratory fitness and weight status would demonstrate higher level of knowledge about physical activity and fitness, more positive attitude toward PE, and higher level of physical activity but lower level of sedentary behavior.

## Methods

### Setting and participants

Data were collected from a public middle school in Iowa, U.S.. The school enrolled 571 students (5-8th grades) at the time of this study (it’s typical for middle or junior high schools in the state to enroll 5th grade students). The majority of the students were non-Hispanic White (85.6%) with a significant ratio of them (40.5%) eligible for free (*n* = 194) or reduced lunch (*n* = 37) and a 12.43 pupil/teacher ratio. PE instructional facility included a multi-purpose gymnasium, a matted wrestling room, a well-equipped weight room, and a 400-m outdoor track. The teachers followed the multi-activity PE curriculum that offered short units of assorted physical activities (e.g., sports, fitness, games, etc.).

All students in 6th, 7th, and 8th grades were eligible for participation in the study and a total of 343 (out of 443 students, 77.4% participation rate) students provided intact data for all measures. The final sample consisted of 6th (*n* = 95), 7th (*n* = 128), and 8th grades (*n* = 120) students whose average age was 12.76 (SD = .94, ranging from 11 to 14) years old. The sample was roughly even by gender (Boys: *n* = 164, 47.8%) with White being the predominant racial group (*n* = 244, 84.5%), which was typical in the state. The Iowa State University Institutional Review Board (IRB) and the school district approved of the study. The study was conducted as a natural process of the participating school’s PE program. Approved by the school’s administrators and the PE teachers, the data collection protocol was integrated into the school’s PE curriculum schedule. Research findings were shared with the PE teachers as feedback to their program evaluation. Results based on de-identified data were authorized for dissemination beyond the school. Due to the participatory research nature of this study, parental consent was waived.

### Variables and instruments

#### Knowledge of physical activity and fitness (PAF knowledge)

PAF knowledge was assessed by the questions for middle school students from the *PE Metrics*™ [31]. The PAF knowledge test includes 29 multiple-choice questions with four possible choices (i.e., A, B, C, and D) which was previously validated using the Rasch Model analysis. The students’ responses were scored as *1 (correct)* or *0 (incorrect)* following the answer key. One question, for example, is phrased as: *Mary performs stretching exercises and runs most days of the week to be able to increase her:* A. *Arm and shoulder strength.* B. *Muscle endurance and abdominal strength.* C. *Flexibility and aerobic endurance* [correct answer]. D. *Flexibility and body weight.* Percentage of correct scores for each participant was computed and used in the data analyses.

#### Attitude toward PE

The *Attitude toward Physical Education Questionnaire for Middle School Students* was used to measure students’ attitude [32]. The instrument contains 20 questions with 10 items capturing the affective component of attitude labeled as “perceived enjoyment” and the other 10 capturing the cognitive component of attitude labeled as “perceived usefulness”. For example, one question asking about students’ perceived usefulness is phrased as “*The games I learn in my physical education class seem important to me”*. The questionnaire was previously validated using middle school samples [29, 32]. All of the items used the 5-point Likert scale for responses ranging from *1 (strongly disagree)* to *5 (strongly agree)*. The questionnaire showed very good internal consistency for “perceived enjoyment” (Cronbach’s *α* = .89) and “perceived usefulness” (Cronbach’s *α* = .86) in the present sample.

#### Physical activity and sedentary behavior

The Youth Activity Profile (YAP) was used to measure students’ physical activity and sedentary behavior [33]. The questionnaire was previously validated with a middle school student sample and showed highly accurate group-level estimates of time spent in physical activity and sedentary behavior [33]. One item measuring sedentary behavior is stated as: *How much time did you spend watching TV outside of school time?* YAP has 15 questions measuring students’ physical activity at school, physical activity after school, and sedentary behavior.

#### Cardiorespiratory fitness and weight status

Cardiorespiratory fitness in this study was assessed by the 20-m PACER (FitnessGram; Cooper Institute 2010). PACER laps were converted into VO2max using the following predictive eq. [34]: *VO2max = 41.77* *±* (PACERlaps * 0.49) - 0.0029 * (PACERlaps^2) - 0.62 * BMI *±* 0.35* Age * Gender. Body mass index (BMI) computed from weight and height was used to define weight status. Weight and height were assessed using a digital weight scale (Tanita HD-366; Tanita, Arlington Heights, IL, USA) and the Seca 213 stadiometer (Seca, Hanover, MD, USDA). Following the FitnessGram standards chart (version 9), raw scores from both VO^2^max and BMI were converted into achievement of the HFZ (1 = in HFZ; 0 = not in HFZ). As two independent variables, cardiorespiratory fitness and weight status served as categorical variables (fixed factors) in this study. FitnessGram has shown sound validity, reliability, and practicality in school PE [34, 35].

### Data collection and processing

The data collection of the study was conducted across the 2016–2017 academic school year. Two separate batches of surveys were administered to collect demographic variables, PAF knowledge, attitude toward PE, physical activity and sedentary behavior. FitnessGram tests were administered by the PE teachers with assistance of trained data collectors. PACER is a commonly used field test in schools. The PE teachers had previously been trained to administer the test and the students were familiar with the testing procedure. To ensure data quality, a hard copy of the testing manual for PACER was delivered to the PE teachers two weeks before data collection started. The trained data collectors measured the students’ weight and height with adequate privacy and confidentiality ensured in the adjacent wrestling room. After data collection was completed, data derived from the two surveys and FitnessGram tests were entered into a Microsoft Excel spreadsheet and then a SPSS24.0 database. We matched data by students’ lunch identification numbers, which were destroyed after data were matched to maintain anonymity.

### Data analysis

Descriptive analysis (mean, standard deviation) and partial correlational analysis (gender and grade as covariates) was conducted for the key variables (attitude, knowledge, physical activity and sedentary behavior), followed by inferential statistical analysis to explore group differences by gender and grade. We tested gender- and grade-based differences to identify whether they would be confounding variables for the subsequent analysis. MANCOVA was conducted to test the effects of cardiorespiratory fitness and weight status on PAF knowledge, attitude toward PE, physical activity and sedentary behavior, after controlling for gender and grade. Data were screened for violations of statistical assumptions (Box’s M test for MANOVA and Levene’s test for ANOVAs). An alpha level of .05 was used to determine significance level and partial eta square (${\eta}_p^2$) was reported for effect size.

## Results

Table 1 shows the descriptive analysis results for the key variables by gender and grade. For gender difference, girls on average showed higher PAF knowledge than boys (*M =* 52.9% vs 49.3%), but lower levels of attitude toward PE (*M =* 70.34 vs 74.94) and physical activity (*M =* 3.31 vs 3.51). These differences were statistically significant (*p* < .05). Sedentary behavior level was similar between girls and boys (*M =* 2.56 vs 2.48). For group differences by grade, students in 6th and 7th grades on average showed a higher level of knowledge than those in 8th grade (6th grade: *M =* 52.0%, 7th grade: *M =* 55.0%, 8th grade: *M =* 46.5%). Students in lower grades showed more positive attitude toward PE (6th grade: *M =* 74.65, 7th grade: *M =* 73.38, 8th grade: *M =* 69.73) and lower sedentary behaviors than students in higher grades (6th grade: *M =* 2.22, 7th grade: *M =* 2.60, 8th grade: *M =* 2.69). These differences were statistically significant (*p* < .05). No grade differences were found for self-reported physical activity (6th grade *M* = 3.28, 7th grade: *M =* 3.47, 8th grade: *M =* 3.42).

**Table 1:** Descriptive Results of the Variables – Mean (Standard Deviation) and Percentage (%)

Variables	PAF Knowledge	Attitude	PA	SB	PACER Laps	PACER HFZ%	BMI	BMI HFZ%
*Gender*								
Boys	49%(18%)	74.94 (12.62)**	3.51(.77)*	2.48(.69)	35.59 (19.95)**	54.8%	22.56 (5.29)	44.2%
Girls	53%(14%)	70.34 (13.47)**	3.31(.82)*	2.56(.83)	25.80 (15.06)**	63.9%	23.07 (5.60)	49.7%
*Grade*								
6th G	52%(15%)	74.65 (11.24)^a^	3.28(.92)	2.22(.75)^ab^	26.63 (15.53)	56.7%	21.99 (5.38)	47.8%
7th G	55%(15%)^a^	73.38 (13.28)	3.47(.78)	2.60(.78)^a^	30.62 (18.73)	61.0%	22.81 (5.97)	44.7%
8th G	47%(18%)^a^	69.73 (14.41)^a^	3.42(.72)	2.69(.70)^b^	33.54 (19.22)	60.4%	23.51 (4.85)	49.1%

*PAF knowledge* physical activity and fitness knowledge, *PA* physical activity, *SB* sedentary behavior, *PACER* progressive aerobic cardiovascular endurance run, *HFZ* healthy fitness zone, *BMI* body mass index. *p < .05; **p < .01. ^a^denotes significant difference (*p* < .05) between two groups; ^b^denotes significant difference (*p* < .05) between the other two groups

Table 2 shows partial correlation coefficients between the variables after controlling for gender and grade. Attitude is positively correlated with PAF knowledge (*r* = .13, *p* < .05) and physical activity (*r* = .27, *p* < .01), while cardiorespiratory fitness is positively correlated with PAF knowledge (*r* = .20, *p* < .01) but negatively correlated with sedentary behavior (*r* = −.14, *p* < .05). Physical activity and sedentary behavior are reversely correlated (*r* = −.22, *p* < .01). Table 3 shows the results from MANCOVA. There were 59.6% students who achieved the HFZ for cardiorespiratory fitness and 47.1% for weight status. The two-way MANOVA examining the differences between those in HFZ and those not in HFZ for cardiorespiratory fitness and weight status showed unequal covariance matrices (Box’s *M* = 55.72, *F*_30,7510_ = 1.74, *p* = .007), therefore Pillai’s Trace (Λ_Pillai_) values were adopted for the results [36]. The analysis showed that there were significant cardiorespiratory fitness effect on the outcome variables (Λ_Pilla_ = .07, *F*_4,255_ = 5.03, *p* = .001, ${\eta}_p^2$ = .07), but no significant effect for weight status (*p* = .57) in this study, after controlling for gender and grade. Subsequent tests of between-subjects effects showed that PAF knowledge (*F*_1,258_ = 9.49, *p* < .01, ${\eta}_p^2$= .04), attitude toward PE (*F*_1,258_ = 4.45, *p* < .05, ${\eta}_p^2$= .02) and sedentary behavior (*F*_1,258_ = 6.89, *p* < .01, ${\eta}_p^2$= .03) were statistically different between students in HFZ group and those not for cardiorespiratory fitness, all favoring the in HFZ group. There were no significant cardiorespiratory fitness by weight status interaction effects (*p*s > .05).

**Table 2:** Partial Correlation Matrix between the Variables (Gender and Grade as Covariates)

	1	2	3	4	5
1. PAF Knowledge					
2. Attitude	.13*				
3. PA	.02	.27**			
4. SB	.02	−.12	−.22**		
5. HFZ.PACER	.20**	.12	.10	−.14*	
6. HFZ.BMI	.10	.08	.00	−.02	.53

*PAF knowledge* physical activity and fitness knowledge, *PA* physical activity, *SB* sedentary behavior, *PACER* progressive aerobic cardiovascular endurance run, *HFZ* healthy fitness zone, *BMI* body mass index. *p < .05; **p < .01

**Table 3:** Two-Way (Fitness and Weight status) MANCOVA (Gender and Grade as Covariates) Results

Source	Variables	*SS*	*df*	*MS*	*F*	*p*	${\eta}_p^2$
Intercept	PAF Knowledge	1.39	1	1.39	57.95	.00	.18
	Attitude	27,172.76	1	27,172.76	166.65	.00	.39
	PA	35.36	1	35.36	60.10	.00	.19
	SB	3.63	1	3.63	7.61	.01	.03
Gender	PAF Knowledge	.01	1	.01	.42	.52	.00
	Attitude	1648.77	1	1648.77	10.11	.00	.04
	PA	2.78	1	2.78	4.73	.03	.02
	SB	1.57	1	1.57	3.30	.07	.01
Grade	PAF Knowledge	.13	1	.13	5.37	.02	.02
	Attitude	989.27	1	989.27	6.07	.01	.02
	PA	.15	1	.15	.26	.61	.00
	SB	4.78	1	4.78	10.03	.00	.04
HFZ.PACER	PAF Knowledge	.23	1	.23	9.49	.00	.04
	Attitude	726.06	1	726.06	4.45	.04	.02
	PA	1.74	1	1.74	2.97	.09	.01
	SB	3.29	1	3.29	6.89	.01	.03
HFZ.BMI	PAF Knowledge	.01	1	.01	.50	.48	.00
	Attitude	68.91	1	68.91	.42	.52	.00
	PA	.47	1	.47	.80	.37	.00
	SB	.89	1	.89	1.86	.17	.01
HFZ.PACER * HFZ.BMI	PAF Knowledge	.03	1	.03	1.12	.29	.00
	Attitude	465.73	1	465.73	2.86	.09	.01
	PA	.00	1	.00	.00	.98	.00
	SB	.44	1	.44	.93	.33	.00
Error	PAF Knowledge	6.20	258	.02			
	Attitude	42,068.51	258	163.06			
	PA	151.81	258	.59			
	SB	123.03	258	.48			
Total	PAF Knowledge	6.64	263				
	Attitude	45,685.97	263				
	PA	156.64	263				
	SB	132.84	263				

*PAF knowledge* physical activity and fitness knowledge, *PA* physical activity, *SB* sedentary behavior, *PACER* progressive aerobic cardiovascular endurance run, *HFZ* healthy fitness zone, *BMI* body mass index. *SS* sum of squares, *df* degrees of freedom, *MS* mean of squares, *F* F statistic from the MANCOVA, ${\eta}_p^2$ = partial eta square

## Discussion

The purpose of this study was to examine the effects of cardiorespiratory fitness and weight status on middle school students’ PAF knowledge, attitude toward PE, and physical activity and sedentary behavior. The hypothesis was partially confirmed in that students with healthy fitness (cardiorespiratory fitness in HFZ) showed significantly higher level of PAF knowledge, more positive attitude, and lower level of sedentary behavior than those with unhealthy cardiorespiratory fitness (not in HFZ), but weight status did not show significant effect on these variables. The results and implications are discussed below.

The results indicate that gender and grade are two demographic variables that are significantly associated with PAF knowledge, attitude, and behavior. Therefore, these two variables were controlled for in the MANCOVA when examining the effects of cardiorespiratory fitness and weight status on PAF knowledge, attitude, and behaviors. The findings suggest physical educators may structure gender sensitive and developmentally appropriate fitness education to cater for the needs and physical activity/fitness preferences of boys and girls in middle school years. Prior research has pointed out that boys and girls at different grade levels tend to have distinct attitudinal preferences when it comes to selection of physical activities for participation [37, 38]. Fitness activities/games, that fit the needs and preferences of boys and girls and that factor in students’ age/grade characteristics, will motivate students to exhibit active participation in these activities both in and outside of PE classes, which will ultimately increase the chance for bettering fitness and adopt the lifestyle of active-living.

The main finding of this study lies in that students who are physically fit (in HFZ for cardiorespiratory fitness) have shown higher level of PAF knowledge, more positive attitude towards PE, and have engaged in less sedentary behavior than those who are physically unfit, regardless of their weight status. This finding is encouraging, as middle school students’ attitude toward PE declines by age [29] and they have shown deficiency about health-related knowledge (e.g., health-related fitness, energy balance, exercising) [19, 30] and spend a significant amount of time being sedentary (e.g., screen time) [5, 6]. Holding a positive attitude toward PE and physical activity experiences and achieving a solid understanding about PAF knowledge are significant correlates of physical activity [20, 39, 40]. Therefore, purposeful fitness education with focus on enhancing cardiorespiratory fitness should be instituted in middle school PE classrooms for students to pursue knowledge, attitude, and behaviors needed for active-living [9, 11]. Exercises or physical activities of moderate (≥ 3 METS) to vigorous intensities (≥ 6 METS) for a minimum of 10 min per bout are beneficial to the cardiorespiratory system, which if practiced regularly will improve or sustain one’s cardiorespiratory fitness [15, 16, 24]. PE teachers are suggested to allot a significant duration of class time in each PE lesson on conventional aerobic exercises such as running, cycling, swimming, active sports and/or novel exercises such as high intensity interval training (HIIT) [41]. In addition, they should encourage students to engage in these activities outside of school, because adequate practice and participation are required to improve fitness [11, 42].

The findings of this study also confirm a similar conclusion observed in epidemiology research in that despite one’s weight status, fitness is an important factor underlying various outcomes in adults and youth populations [15, 16, 24]. Prior research indicate that a good level of cardiorespiratory fitness may offset the hazardous effects of weight status [15, 16, 24]. Our study reinforces the importance of conveying purposeful fitness education through middle school PE, while also addressing body composition (learning knowledge, strategies, and skills pertinent to weight management) as a secondary goal. Although it remains an important goal of fitness education to achieve a healthy body composition, to change weight or body composition is difficult and requires synergistic efforts from multiple layers of environmental factors and components [24]. The findings are promising for middle school students, especially for those who are unable to maintain a healthy weight during adolescence years. Our study did not observe any significant group differences in any of the focal variables (knowledge, attitude, physical activity or sedentary behavior) between the healthy weight group and the need improvement weight group. In other words, weight status does not necessarily interfere with students’ learning processes and outcomes in PE classes (e.g., to gain a positive attitude, acquire knowledge, and participate in physical activities). With increased engagement and learning in PE classes, students will likely enhance fitness, which will likely lead to more desirable outcomes for attitude, knowledge learning, and sedentary behavior. Further, sustained engagement in regular physical activities will increase caloric expenditure therefore changes in body composition in the long run [19].

The findings from this study should be interpreted with several limitations. First, this study utilized a correlational design. The significant associations between cardiorespiratory fitness (and weight status) and the key variables should not be interpreted as cause and effect. Second, despite the large sample size, the participants were recruited from one middle school located in a mid-western state. The findings are only generalizable to middle school students in schools of similar characteristics. Last but not the least; our advocacy of focusing cardiorespiratory fitness in school-based fitness education as curriculum priority was based upon the data reported in this study. This advocacy does not suggest that other fitness components are not important content of fitness education.

## Conclusion

This study showed significant effect of cardiorespiratory fitness (not weight status) on PAF knowledge, attitude, and sedentary behavior in middle school students. The findings suggest that achieving the HFZ for cardiorespiratory fitness is associated with higher PAF knowledge, more positive attitude toward PE, and lower level of sedentary behavior. In light of the obesity and physical inactivity epidemic, fitness education with focus on cardiorespiratory fitness should become a curriculum priority in middle school PE classroom.
